# Polyphosphate during the Regreening of *Chlorella vulgaris* under Nitrogen Deficiency

**DOI:** 10.3390/ijms161023355

**Published:** 2015-09-28

**Authors:** Fei-Fei Chu, Xiao-Fei Shen, Paul K. S. Lam, Raymond J. Zeng

**Affiliations:** 1 Advanced Laboratory for Environmental Research and Technology, USTC-CityU, Suzhou 215123, China; E-Mails: chuffei@mail.ustc.edu.cn (F.-F.C.); sxf0912@mail.ustc.edu.cn (X.-F.S.); bhpksl@cityu.edu.hk (P.K.S.L.); 2 College of Standardization, China Jiliang University, Hangzhou 310018, China; 3 State Key Laboratory in Marine Pollution, Department of Biology and Chemistry, City University of Hong Kong, Kowloon 999077, Hong Kong; 4 CAS Key Laboratory of Urban Pollutant Conversion, Department of Chemistry, University of Science and Technology of China, Hefei 230026, China

**Keywords:** *Chlorella vulgaris*, regreening, biodiesel production, polyphosphates, nitrogen deficiency, phosphate supply

## Abstract

Polyphosphate (Poly-P) accumulation has been reported in *Chlorella vulgaris* under nitrogen deficiency conditions with sufficient P supply, and the process has been demonstrated to have great impact on lipid productivity. In this article, the utilization of polyphosphates and the regreening process under N resupplying conditions, especially for lipid production reviving, were investigated. This regreening process was completed within approximately 3–5 days. Polyphosphates were first degraded within 3 days in the regreening process, with and without an external P supply, and the degradation preceded the assimilation of phosphate in the media with an external P offering. Nitrate assimilation was markedly influenced by the starvation of P after polyphosphates were exhausted in the medium without external phosphates, and then the reviving process of biomass and lipid production was strictly impeded. It is, thus, reasonable to assume that simultaneous provision of external N and P is essential for overall biodiesel production revival during the regreening process.

## 1. Introduction

N deficiency or limitation has taken prominence in the last decade due to increased interest in the improvement of the lipid content of microalgal cells [1,2,3,4,5]. Cells usually change their metabolism to proceed to an abiotic status in response to N deficiency; for example, total neutral lipid and carbohydrate content increase rapidly in algal cells as a protective mechanism for surviving in unfavorable conditions, and this mechanism is also termed the “lipid trigger” [3,5]. During this time, cell division ceases, resulting in the lower growth rates and poor lipid productivity [3,6]. However, if N deficiency is applied to stimulate oil accumulation simultaneously with sufficient P supply, lipid productivity is greatly enhanced as compared to that of the nutrient complete media [7,8]. Moreover, P is found to be assimilated luxuriously and accumulates as Poly-P in *C. vulgaris* cells [7].

The accumulation of Poly-P under nutritional conditions unfavorable for growth is found in many microorganisms [9,10,11]. Structurally, inorganic Poly-P is a linear polymer with tens or hundreds of orthophosphate residues linked by phosphoanhydride bonds [9]. Numerous researchers are attempting to understand the functions that Poly-P performs in the cells. Some purified enzymes have been isolated with a catalyzing role which can reversibly convert the terminal phosphate of Poly-P to ATP [9,10]. In brief, the function of energy supply (ATP substitutes) and the phosphate reservoir to physiologically cope with stress conditions for survival is widely acknowledged [10]. Moreover, lipid metabolism is shifted from membrane lipid synthesis to accumulation of neutral lipids, mainly TAGs, which are also deemed as a storage form of carbon and energy to tackle exoteric stresses [5]. Poly-P and lipids associated with energy and nutrient source probably play important roles during the revival of algal cells when a N source is supplied.

Microalgal cells are characterized by the metabolic degradation of proteins and the chloroplasts resulting from N deficiency and, since the loss of photosynthetic capacity follows, the process has previously been termed “degreening” [12]. Correspondingly, the process of “regreening” is the recovery of the photoautotrophic growth capacity of algal cells upon the resupply of N [12,13]. Most studies previously focused on the regreening process mainly in terms of cell differentiation in order to obtain information about algal chloroplast development [13].

Based on the results reported by other researchers, the Poly-Ps accumulate in algal cells and play an important function during the regreening process [12]. However, there are no systematic studies regarding the fate of intracellular Poly-Ps with respect to the variation of lipid productivity during the regreening process. Chlorophyll *a*, an essential kind of photosynthetic pigment, takes responsibility for capturing solar energy to generate continuous power for both cell growth and lipid accumulation [4]. Therefore, chlorophyll *a* content was measured in our study so as to better understand the regreening process and the relationship between Poly-P utilization and lipid production. Moreover, a follow-up degreening stage after the first stage for Poly-P and lipid accumulation was also applied in the present study for comparison.

The goal of the present work was to investigate the fate of Poly-Ps and its relationship with the regreening and degreening processes. Firstly, in what situation could Poly-P be used? Secondly, what is the phosphate utilization order when extracellular phosphate and intracellular Poly-P exist simultaneously? Thirdly, what are the effects of Poly-P usage in the regreening process, especially on lipid production?

## 2. Results and Discussion

### 2.1. Profiles of Biomass Growth and Chlorophyll a

**Figure 1:**
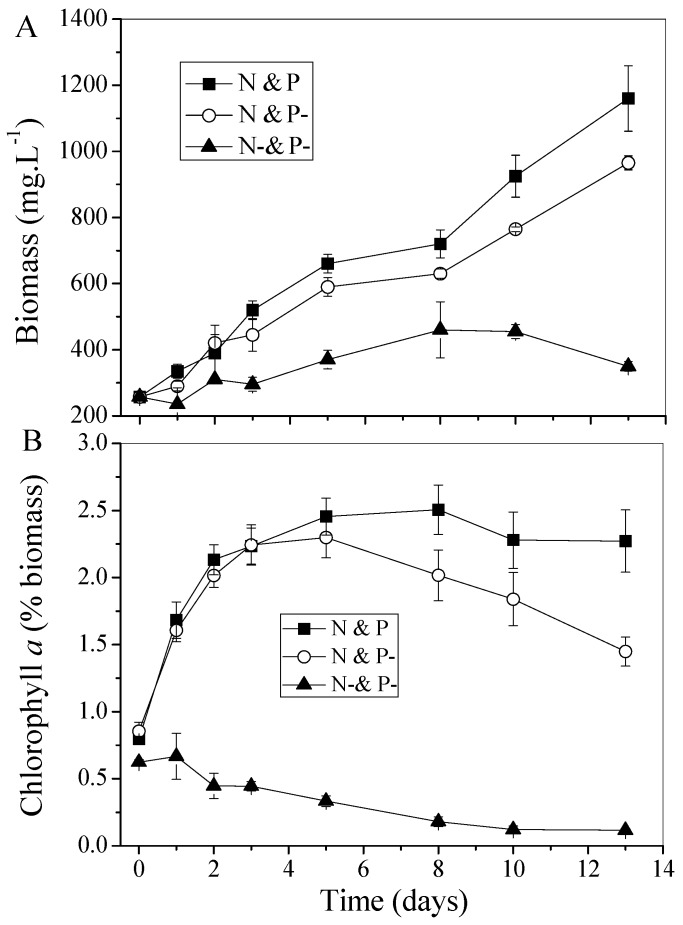
Profiles of (**A**) biomass growth and (**B**) chlorophyll *a* cont**e**nt of *C. vulgaris* in different media. Values shown are averages of two samples ± range.

N deficient algal cells proceed to a photosynthetic active status after being resupplied with nitrate, via the process termed “regreening”, which is characterized by a reassembling of the photosynthetic apparatus, re-synthesizing protein, and by utilization of Poly-P, carbohydrates, and lipids. The regreening and continual degreening processes were investigated with respect to the change of biomass and chlorophyll *a* content. The time-course profiles of biomass in the three media are shown in Figure 1A. The biomass concentration persistently increased in the media with an extracellular N source supply. The maximum biomass of C. *vulgaris* obtained in the N and P condition was approximately 1160 mg·L^−1^, followed by the N and P− condition with a biomass concentration of 965 mg·L**^−^**^1^ after 13 days cultivation. Moreover, the biomass growth rates of N and P and N and P− during the first three days were very similar. However, there was a detrimental effect for algal cells during the continuous N deficient phase. Clearly, biomass increased slowly and the maximum concentration was obtained at the eighth day in the N− and P− condition. The increased biomass may be explained by the accumulation of lipid in algal cells with N starvation stimulation. The culture media were taken regularly for the determination of chlorophyll *a*. As shown in Figure 1B, chlorophyll *a* content in the N− and P− condition dropped from 0.62% to 0.11%, and a similar decrease also occurred in the N and P− condition after a dramatic increase in the initial three days, while chlorophyll *a* content maintained almost unchanged in the N and P condition (approximately at the level of 2.5%) in the latter stage of cultivation.

The optimal conditions for the regreening process are supposed to be offering not only sufficient N but also sufficient P, that is, the N and P condition in this study. It was clear that the regreening process for *C. vulgaris* was completed within 3–5 days, as judged by the change of chlorophyll *a* content which reached its maximum value on the fifth day. However, upon exposure to a N containing medium, cells of *C. fusca* began the regreening period, with chlorophyll synthesis completed within 24 h [13]. The content of chlorophyll *a* in *Phaeodactylum tricornutum* was increased in the third days after the resupply of nitrate [14]. These different lengths of the regreening period are probably due to the different algal species. 

Cell metabolism is induced to change under conditions of N starvation, and the most notable of these changes is the reassembling of the photosynthetic apparatus and the loss of photosynthetic capacity to a level even below that of spores [12]. However, it was obvious that the biomass continually increased after external N was deficient in N− and P− conditions as shown in Figure 1A. This phenomenon is hypothetically supported by the presumption that chlorophyll *a* serves as an intracellular N source when the external N sources are exhausted [4]. Clearly, chlorophyll *a* decreased from 0.62% to 0.11% in the N− and P− condition (Figure 1B). The phenomenon of chlorophyll *a* degradation in the external N unavailable condition was also verified by other researchers. Lv *et al.* [15] reported that chlorophyll *a* content of *C. vulgaris* began to decrease dramatically once nitrate was completely consumed. In another algal species, *Rhodomonas* sp., the content of chlorophyll *a* increased from 0.5 to 1.5 pg/cell in the first N available stage and then decreased to 0.3 pg/cell when the nitrate was exhausted [16]. However, when N−sufficient conditions returned, chlorophyll *a* was again accumulated with the content increasing at a very rapid rate. The chlorophyll *a* content increased obviously from 0.8% to 2.5% during the first 5 days in our experiment and then remained at its maximal level in the N and P conditions, while it dropped sharply at the later stage of N and P−. Revealing the reason for this phenomenon should get to the root of phosphate deficiency. 

### 2.2. Nutrient Assimilation Profile

**Figure 2:**
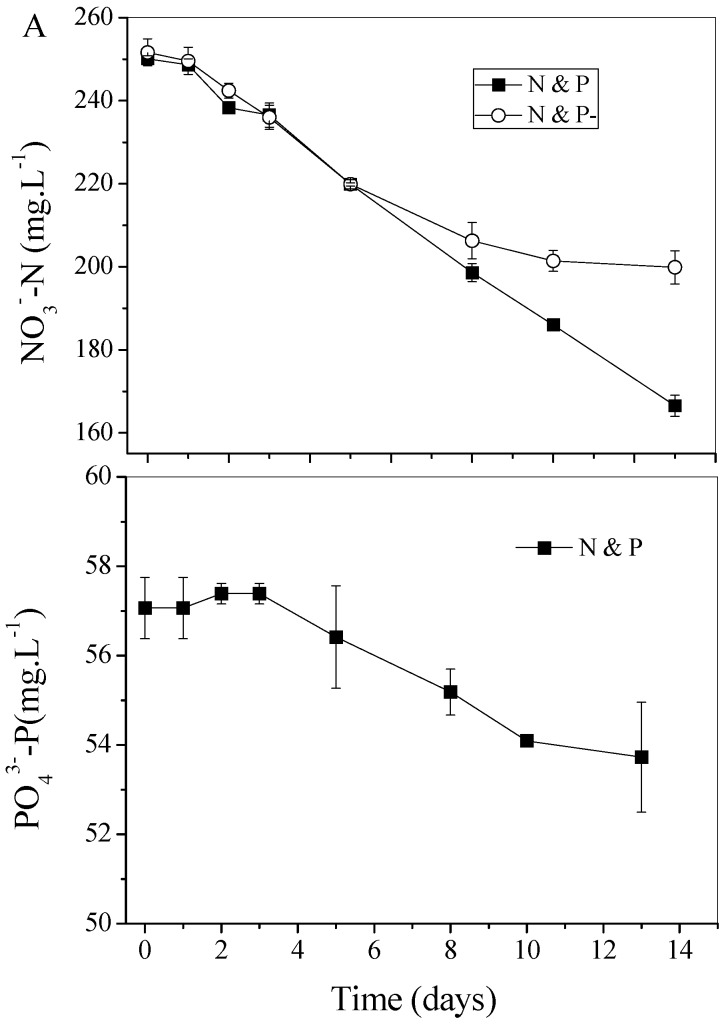
Profiles of (**A**) nitrogen (NO_3_**^−^**-N) and (**B**) orthophosphate (PO_4_^3**−**^-P) assimilation by *C. vulgaris* cultured in different conditions. Values shown are averages of two samples ± range

Figure 2 shows the time-course profiles of the concentration of N (PO_4_^3**−**^-N) and P (PO_4_^3**−**^-P) in different cultivation conditions. As shown in Figure 2A, in the first cultivation stage, the N uptake rate was quite similar in N and P and N and P− conditions, while it gradually slowed down five days later in the N and P− condition. Figure 2B shows the orthophosphate assimilation profile in the N and P condition. No obvious consumption was observed during the first stage of cultivation, but PO_4_^3**−**^-P began to be assimilated after three days cultivation (Figure 2B).

### 2.3. Changes in Storage Products during the Regreening Process

**Figure 3:**
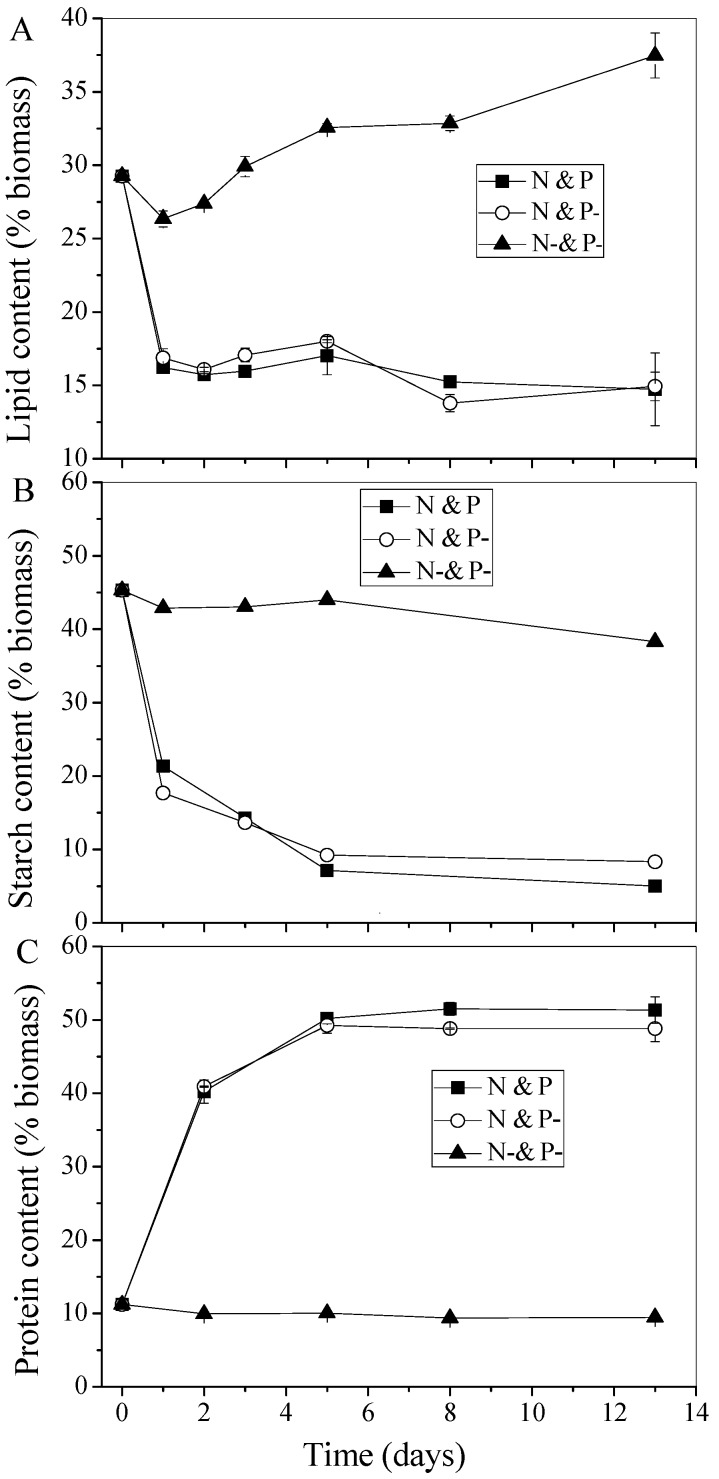
Storage products of *C. vulgaris* grown in different media. (**A**) lipid; (**B**) starch; and (**C**) protein. Values shown are averages of two samples ± range.

To investigate the fate of intracellular Poly-P with respect to the variation of lipid productivity during the regreening process, changes in the contents of the lipids, starch, and protein of *C. vulgaris* over time were determined. As shown in Figure 3A, lipid content decreased slightly in the first day and then increased dramatically from 26.3% to 37.4% in the N− and P− condition, which was in the continual degreening process with increased lipid content. The possible reason for the slight decrease of lipid content in the first day is probably due to self-regulating for the new cultivation condition or can be explained by systematic errors. However, in the regreening process, lipid content decreased rapidly from 39.3% to an average level of 15.8% within 24 hours upon supplying with N, and the low level of 15.8% was the usual lipid content in normal growing cells of *C. vulgaris* [7]. As shown in Figure 3B,C, protein and starch contents remained almost unchanged in N− and P−, which can be explained by the fact that cells were still in the exoteric N depletion stress condition. In terms of the regreening process in N and P and N and P−, the changes of starch and protein contents all lagged behind lipid variation. Starch content decreased rapidly within 5 days and then remained at low levels during the following 8 days with final contents of 5.0% and 8.3% in N and P and N and P−, respectively (Figure 3B). However, the protein content increased dramatically to the normal growth level within 5 days, and the final contents in the N and P and N and P− conditions were 51.3% and 48.8%, respectively.

### 2.4. Microscopic Analysis of Lipid Droplets and Poly-P and ^31^P NMR

**Figure 4:**
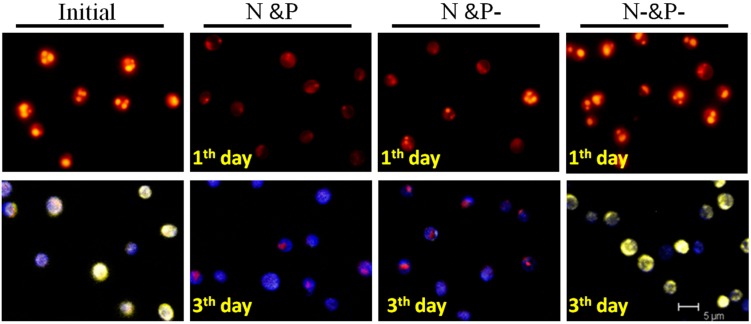
Observation of neutral lipid and Poly-P using Nile Red and DAPI staining. Above: The golden yellow fluorescence in the images indicates the presence of neutral lipid, and the red fluorescence is the autofluorescence of chlorophyll. Below: Blue fluorescence comes from nucleus staining, bright yellow fluorescence shows the Poly-P, and red fluorescence comes from *chlorophyll.*

*C. vulgaris* cells change their metabolic pathways to accumulate a large amount of neutral lipids and Poly-P granules upon stimulation by N starvation [7]. If the cells are suspended in different nutrient conditions, changing profiles of neutral lipids and Poly-P can be monitored by staining with Nile Red and DAPI. As shown in Figure 4, the gold-yellow fluorescence observed indicated that a high content of neutral lipids still existed in cells under the N− and P− condition, while the lipid contents were rather low in N and P and N and P− conditions after one day cultivation. From the DAPI staining images, the bright yellow fluorescence in the initial cells and the cells under N− and P− condition illustrated the existence of high Poly-P content, while it was hardly seen in the three-day cultivation cells under N and P and N and P− conditions. Meanwhile, red fluorescence coming from the chlorophyll *a* indicated that cells gradually accumulated a certain amount of chlorophyll *a* (Figure 4).

**Figure 5:**
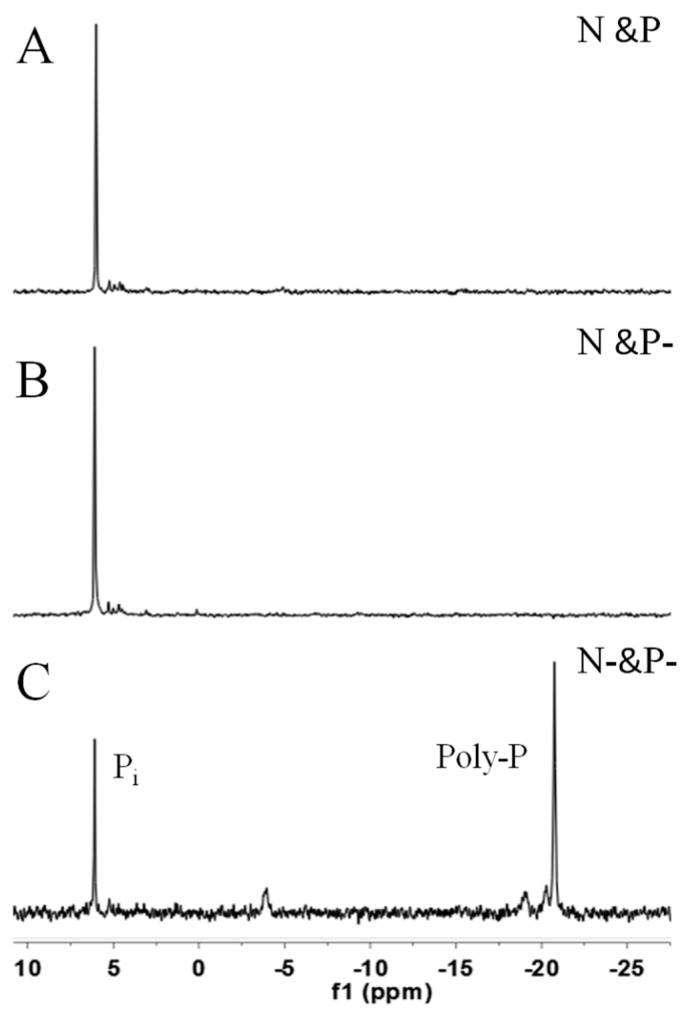
The ^31^P NMR spectra of *C. vulgaris* cultured in N and P (**A**); N and P− (**B**); and N− and P− (**C**) media for three days. P_i_; inorganic phosphate, Poly-P; polyphosphates.

Figure 5 shows NMR spectra of *C. vulgaris* in N and P (A), N and P− (B), and N− and P− (C) conditions after three-day cultivation. The intense signal of the mid-chain P groups of Poly-P at −21 ppm illustrated that the algal cells at N− and P− still had high amounts of Poly-P, while the distinct peak at approximately 6.5 ppm showed that inorganic orthophosphate were very abundant in the cells of the three cultivation conditions.

Poly-P, judged by its structure, can serve as a phosphate/energy source and plays a significant role during the regreening process where the cells recover when the external conditions once again favor cell growth [12]. The intense signal of the mid-chain P groups of Poly-P shown in Figure 5C indicated that, as compared with N resupply conditions, Poly-P could hardly be utilized smoothly in N− and P− conditions. This phenomenon leads to the conclusion that the prerequisite for Poly-P usage is the existence of N, since Poly-P is also proven as a regulator for stress and survival [10]. Nevertheless, Poly-P could almost not be found in cells in the N and P and N and P− conditions after 3 days cultivation based on the signals shown in ^31^P NMR (Figure 5A,B) and DAPI staining images (Figure 4). The data presented in Figure 2B show that orthophosphate was not assimilated until the fourth day in the N and P condition. It therefore seemed more reasonable to assume that Poly-P was obligatory during the first stage of regreening progress. Similarly, Kuesel *et al*. [12] noted that *C. fusca* cells contained the same amount of Poly-P in both phosphate supplied and unsupplied conditions after 8 h regreening, which indicated that Poly-P, as an important phosphate source, is utilized first during the first period of the regreening process.

The functioning of Poly-P as an energy and phosphate source is widely acknowledged. In many organisms, several enzymes are involved in the process to catalyze the transfer of energy and Pi among AMP, ATP and Poly-P [9,10]. For example, Poly-P kinase and AMP−phosphotransferase are two important enzymes necessary for the energy release from Poly-P [10]. Polyphosphatases, the enzymes catalyzing the hydrolysis of Poly-P to Pi, are responsible for offering a phosphate source via Poly-P [9,17].

### 2.5. Productivity of FAMEs

**Figure 6:**
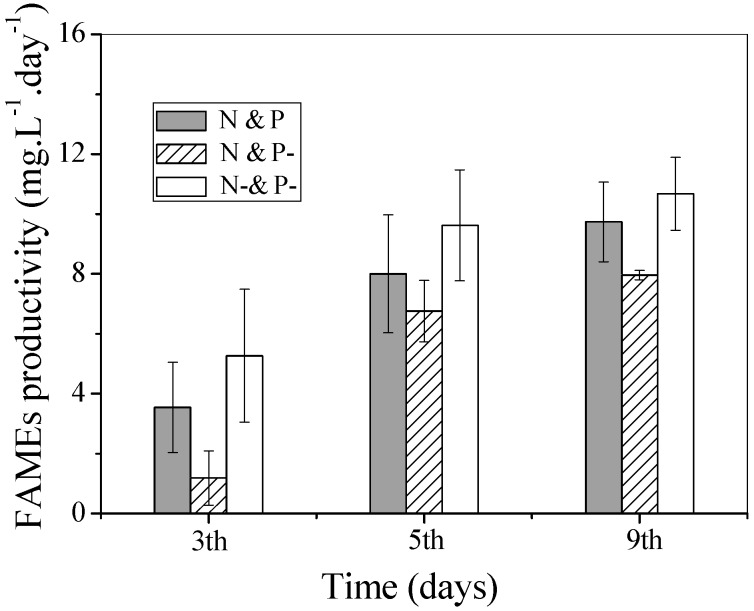
FAME productivity of *C. vulgaris* cultured in N and P, N and P−, and N− and P− media. Values shown are averages of two samples ± range.

Productivity of FAMEs is an important indicator of biodiesel producing capacity, and can be calculated from the FAME content and biomass productivity. The time-course profiles of FAME productivity in the three culture conditions are shown in Figure 6. FAME productivity increased gradually during the degreening and regreening processes. Relatively higher FAME productivity was observed in the N− and P− culture and the highest was 10.7 mg·L**^−^**^1^·day**^−^**^1^ at day 9, which was attributed to the higher FAME content. In the regreening process, regarding the effect of external P, the slightly higher FAME productivity in the N and P as compared with the N and P− cultures indicated the positive effect of P on biodiesel production.

### 2.6. Overall Analysis of this Work

The fact that the rapid consumption of Poly-P in N and P and N and P− conditions indicated that Poly-P could only satisfy the needs of regreening cells for a very short time and, without external phosphate replenishment, the algal cells would be in a state of P depletion. The nitrate assimilation profile in Figure 2A clearly showed that the process of N consumption was strictly influenced by P starvation in N and P− conditions after 5 days of cultivation. Then, algal cells in this condition gradually entered another state with the simultaneous absence of N and P. Chlorophyll *a*, an N− rich compound, was then degraded to offer endogenous N as shown in the N and P− condition. Without the high photosynthetic activity obtained from chlorophyll, the biomass growth lagged behind with the lower growth rate in N and P− as compared with N and P condition. Undoubtedly, in terms of the lipid reviving process (as shown in Figure 6), FAME productivity was dramatically depressed in N and P− condition by lower biomass productivity in spite of the same lipid content in the two N sufficient conditions. These results revealed that with regard to the regreening process, especially for biomass and lipid production revival, it was necessary to supply external N and P at the same time.

The degreening process for *C. vulgaris* was also studied previously [7]. It was demonstrated that P was assimilated rapidly in the degreening process as compared with nutrients complete condition. However, the stationary phase was not reached after 14 days cultivation in N deficient condition with P sufficient supply [7]. It is reasonable that the late stage of the growth would be N− and P− condition after P was assimilated completely in the previous study. Obviously, algae cells got into stationary phase after a slow growth stage for 8 days (Figure 1). In this work, the difference of biomass growth rate between N and P and N and P− conditions was smaller than that in our previous study [7]. The reason for this is probably the Poly-P existed in algae cells in N and P− condition. Moreover, the absorption of N nearly ceased in P deficiency condition in our previous study [7], while it only stopped completely after 10 days in N and P− due to the existence of Poly-P as internal P. This phenomenon, once again, demonstrates that the existence of P is the prerequisite for nitrogen utilization for algal cells.

## 3. Experimental Section 

### 3.1. Strains and Culture Conditions

The strain of *C. vulgaris* FACHB-1072 was provided by the Freshwater Algae Culture Collection at the Institute of Hydrobiology (FACHB) in Wuhan, Hubei Province, China. An assembled device for algal cultivation was described in a previous study [7]. BG11 medium and the modified BG11 medium were prepared and autoclaved at 121 °C for 30 min to cultivate *C. vulgaris* FACHB-1072. The temperature was maintained at 25 ± 2 °C. Light intensity was 120 μmol·photons·m^−2^·s^−1^ with a light/dark ratio of 16:8. Mixed gases (4% CO_2_ in air) were bubbled through a 0.20 μm gas filter diaphragm Midisart-2000 (SRP65, Sartorius, Germany) at a flow rate of 0.25 *v*/*v*/min. The initial pH was 6.8.

### 3.2. Experimental Design

*C. vulgaris* FACHB-1072 used for the inoculum had previously been grown in BG11 medium for 6 days to reach a sufficient concentration. Then, the algal cells were centrifuged and re-suspended in modified BG11 medium with the NaNO_3_ being removed. In addition, approximately 80 mg·L**^−^**^1^ PO_4_^3**−**^-P instead of the customary concentration of 5.43 mg·L**^−^**^1^ was added to the modified BG11 media, so as to provide sufficient P to the algal cells for the luxury uptake of P under N−deficient conditions. After cultivation for 14 days under N deficiency, the algal cells were centrifuged again and re-suspended at an initial concentration of 250 mg·L**^−^**^1^ in three types of modified BG11 medium: sufficient nitrogen and phosphorus (N and P), sufficient nitrogen but phosphorus deficient (N and P−), and nitrogen and phosphorus deficient (N− and P−). The initial concentrations of nitrate and orthophosphate for these three media are shown in Table 1. The experiments with two duplicates lasted 14 days. Liquid samples were collected to measure biomass and chlorophyll *a* concentrations. Algal cells were collected and converted into algal powder after being freeze-dried. Subsequently, the contents of lipid, carbohydrate, and protein were determined. Poly-P was characterized using confocal laser scanning microscopy and ^31^P NMR.

**Table 1:** The initial concentrations of nitrate and orthophosphate for three types of culture media.

Initial Concentration	N and P	N and P−	N− and P−
N (NO_3_^−^-N) (mg·L^−^^1^)	250	250	0
P (PO_4_^3−^-P) (mg·L^−^^1^)	57	0	0

### 3.3. Analysis

#### 3.3.1. Growth Parameters

The microalgal biomass was determined using the standard method of suspended solid measurement [18]. Orthophosphate (PO_4_^3**−**^-P) in the medium was determined using a water quality autoanalyzer (Aquakam 200, ThermoFisher, Vantaa, Finland) following standard methods [18].

#### 3.3.2. Determination of Chlorophyll *a*

Chlorophyll *a* was monitored following the Chinese State Standard Monitoring Methods (Monitoring Methods for Water and Wastewater, 2002) with some modifications: approximately 5 mL of algal suspension was filtered through 0.45 μm mixed cellulose ester membrane, the membrane was then deposited in a tube, which underwent three freezing-thawing cycles at −60 °C and in a 50 °C water bath, before 9 mL of acetone (50 °C) was added into each tube. Following vigorous agitation, all tubes were kept in the dark for about 2.5 h. Distilled water (1 mL) was added into each tube before measurement. The liquid supernatant was collected, and OD_630_, OD_645_, OD_663_, and OD_750_ were measured using a 1 cm cuvette and a UV-VIS spectrophotometer (SHIMADU UV1800, Kyoto, Japan). The content of chlorophyll *a* was calculated based on Equation (1):

(1) $$\text{Chl }a\;\left(\text{mg}.{\text{L}}^{-1}\right)=\frac{10\times \left[11.64\times \left({\text{OD}}_{663}-{\text{OD}}_{750}\right)-2.16\times \left({\text{OD}}_{645}-{\text{OD}}_{750}\right)+0.1\left({\text{OD}}_{630}-{\text{OD}}_{750}\right)\right]}{\text{v}}$$

(Note: where v = sample volume (mL)).

#### 3.3.3. Determination of Biodiesel Production

Biodiesel was quantified as FAMEs after conversion from fatty acids following the direct transesterification method adapted from Rodriguez-Ruiz *et al.* [19]. The transesterification reagent was prepared by mixing acetyl chloride and methanol at a volume ratio of 1:9. Then, the reagent (2 mL) was mixed with 20 mg lyophilized algal powder to obtain the FAMEs, which were subsequently analyzed using a gas chromatograph (Agilent 6890, Santa Clara, CA, USA). The detailed steps are presented in our previous study [7].

#### 3.3.4. Determination of Starch Content

The algal cell starch content was measured using a commercial enzymatic Starch Assay Kit (SA-20, Sigma-Aldrich, St. Louis, MO, USA), and all steps were operated strictly in accordance with the technical instruction.

#### 3.3.5. Determination of Crude Protein Content

The crude protein was determined following the method reported by Becker [20]. Briefly, an equivalence relationship exists between the crude protein content and the N content in algal cells; that is, the “protein content = N content × 6.25”. The N content was determined using an elemental analyzer (Elementar Vario MACEO, Hessia, Germany).

#### 3.3.6. Characterization of Poly-P

The utilization of Poly-P was observed using fluorescence microscopy and ^31^P NMR measurement. For DAPI staining, cells were washed with PBS buffer after centrifugation and then fixed for 30 min with 4% paraformaldehyde in PBS. The cells were centrifuged again and then resuspended in PBS before observation. Briefly, 5 μL of fixed microalgal suspension was collected and placed on a slide, then 1.5 μL DAPI (50 μg/mL) was added and the slide incubated for 10 min in the dark. The samples were observed using a confocal laser scanning microscope (TSC-SP2, Leica, Germany) equipped with a semiconductor laser of λ = 405 nm. For the detection of Poly-P, the emission spectral range 530–540 nm was selected.

For ^31^P NMR determination, the samples were prepared following the perchloric acid extraction method described by Moreno [21]. Nuclear magnetic resonance spectroscopy signals were obtained using a Varian NMR spectrometer (Vnmrs-300, Palo Alto, CA, USA). All the details are described in our previous study [7].

#### 3.3.7. Characterization of Neutral Lipid Drops

The dye Nile Red is widely used to measure the neutral lipid content in microalgal cells [22]. Briefly, an algal suspension was collected and diluted to a lower concentration (OD < 1) with distilled water. After that, 50 μL Nile Red solution (1 mg·mL^−1^ in DSMO) was mixed with 5 mL diluted algal suspension and then incubated in the dark for 1–2 h at room temperature. Neutral lipid staining microscopy was operated with an Olympus BX51 reflection fluorescence microscope (Olympus BX51, Tokyo, Japan) using U-MWIG2 spectroscope components and a 120×/1.25 oil immersion objective.

## 4. Conclusions

This study investigated the utilization of Poly-Ps and biodiesel revival in the regreening process of *C. vulgaris*. This regreening process was completed within approximately 3–5 days in N resupplying media. The fact that Poly-Ps were not utilized in the N− and P− condition indicated that the existence of N was a prerequisite for Poly-P degradation. With simultaneous existence of Poly-P and external phosphate, Poly-P was first degraded within 3 days in the N and P and N and P− conditions and also prior to the assimilation of external P. The decreased nitrate assimilation rate in the later stage of the N and P− condition was likely due to the exhaustion of phosphate, so that the biomass and biodiesel production revival process was strictly hindered. It is therefore reasonable to speculate that a simultaneous supply of external N and P is essential for overall biodiesel production revival during the regreening process.
